# Evaluation of the control efficacy of antagonistic bacteria from V-Ti magnetite mine tailings on kiwifruit brown spots in pot and field experiments

**DOI:** 10.3389/fmicb.2024.1280333

**Published:** 2024-03-12

**Authors:** Yongliang Cui, Yuhang Zhu, Guanyong Dong, Yanmei Li, Jing Xu, Zuqiang Cheng, Lijun Li, Guoshu Gong, Xiumei Yu

**Affiliations:** ^1^Sichuan Provincial Academy of Natural Resource Sciences, Chengdu, China; ^2^Wild Plants Sharing and Service Platform of Sichuan Province, Chengdu, China; ^3^College of Resources and College of Agronomy, Sichuan Agricultural University, Chengdu, China; ^4^Kiwifruit Industry Development Bureau of Cangxi, Guangyuan, China

**Keywords:** biological control, kiwifruit disease, *Bacillus*, inhibitory effect, field efficacy

## Abstract

Seemingly barren heavy-metal-polluted vanadium (V) and titanium (Ti) magnetite mine tailings contain various functional microbes, yet it is unclear whether this includes microbial resources relevant to the biological control of plant diseases. Kiwifruit brown leaf spot disease, caused by *Corynespora cassiicola*, can seriously reduce kiwifruit yield. To discover effective control measures for kiwifruit leaf spot, 18 bacteria strains among 136 tailing-isolated bacteria from V-Ti magnetite mine tailings were identified as inhibiting *C. cassiicola* by the confrontation plate method, indicating that antagonistic bacteria surviving in the V-Ti magnetite mine tailings were present at a low level. The 18 antagonistic strains could be divided into two BOX-A1R clusters. The 13 representative strains that were selected for phylogenetic tree construction based on their 16S rRNA sequences belonged to the *Bacillus* genus. Five predominant strains exhibited different toxin-production times and intensities, with four of them initiating toxin production at 32 h. Among them, *Bacillus* sp. KT-10 displayed the highest bacteriostatic rate (100%), with a 37.5% growth inhibition rate and an antagonistic band of 3.2 cm against *C. cassiicola*. *Bacillus* sp. KT10 also showed a significant inhibitory effect against the expansion speed of kiwifruit brown spots in the pot. The relative control effect was 78.48 and 83.89% at 7 days after the first and last spraying of KT-10 dilution, respectively, confirming a good effect of KT-10 on kiwifruit brown leaf spots in the field. This study demonstrated for the first time that there are some antagonistic bacteria to pathogenic *C. cassiicola* in V-Ti magnetite mine tailings, and *Bacillus* sp. KT10 was found to have a good control effect on kiwifruit brown leaf spots in pots and fields, which provided an effective biological control measurement for kiwifruit brown leaf spots.

## Introduction

Kiwifruit (*Actinidia chinensis*) is a perennial deciduous vine in the family *Actinidiaceae* (Richardson et al., 2018). Kiwifruit is deeply loved by consumers and has broad market prospects, owing in part to its richness in vitamins A, C, E, B6, and B12 and its overall high nutritional value (Drummond, 2013). The wild resources and cultivated areas for kiwifruit in China are considerable, with the main production areas concentrated in Sichuan and Shaanxi Provinces. In Sichuan, kiwifruit is grown extensively around cities like Chengdu, Ya'an, and Guangyuan. In recent years, the cultivated areas have expanded in response to increasing market demand. The total kiwifruit cultivation area in China is 2.43 × 10^5^ hm^2^, yielding an annual harvest of 2.5 × 10^6^ t (Wang et al., 2021). Furthermore, kiwifruit has played a positive role in helping farmers overcome poverty (Richardson et al., 2018).

However, kiwifruit diseases have become increasingly serious with the rapid growth of its cultivation, which is severely affecting kiwifruit yield and quality, leading to substantial economic losses and making the expansion of kiwifruit cultivation one of the main factors restricting the continued development of kiwifruit cultivation. More than 30 kinds of kiwifruit diseases exist, including bacterial canker disease, brown spot, root rot, flower rot, fruit soft rot, phytophthora, leaf blight, root-knot nematode disease, black spot, and other common and severe diseases (Jeong et al., 2008). Kiwifruit brown spot caused by the fungal pathogen *Corynespora cassiicola* is particularly problematic in Sichuan, China, occurring and spreading rapidly in July and August in particular (Cui et al., 2015). It leads to leaf desiccation and early leaf abscission, with high disease rates of 60%−100%, thus adversely affecting photosynthesis, causing branches to wither, fruit loss, and yield reductions of 30%−50%. Thus, the kiwifruit brown spot has become a significant obstacle to the kiwifruit industry's growth (Cui et al., 2015; Zhang et al., 2021).

Hence, there is an urgent need to find more effective biological control methods. Biological control often involves using antagonistic bacteria, metabolites of antagonistic bacteria, and/or plant extracts to control diseases. Many studies have shown that beneficial microbial preparations can indeed effectively control diseases and reduce or even eliminate pesticide residues (Ren et al., 2010). Biological management of kiwifruit postharvest diseases using antagonistic yeasts and bacteria has been demonstrated to be safe and effective (Li et al., 2022). *Candida oleophila*, originally isolated from the surface of tomato fruit, was shown to effectively inhibit gray mold and black rot in kiwifruit caused by *Botryis cinerea* and *Alternaria alternata*, as well as the incidence of natural decay (Wang Y. et al., 2018). Another antagonistic yeast *Meyerozyma caribbia* isolated from the surface of kiwifruit had antagonism to *Penicillium expansum*, resulting in kiwi fruit decay, so it can be used to control postharvest blue mold of kiwifruit (Qiu et al., 2022). Five endophytic bacteria from *Leptospermum scoparium* showed antagonism against *Pseudomonas syringae* pv. *actinidiae* (Psa), the causative agent of kiwifruit canker. Furthermore, three *Pseudomonas* sp. strains of these endophytic bacteria were transmissible to kiwifruit by wound inoculation, where they inhibited colonization by Psa and reduced canker disease severity (Wicaksono et al., 2018). However, there is limited research on the biological control of the kiwifruit brown spots. As such, the identification and development of biocontrol strains for kiwifruit brown spots are of great importance.

The vanadium (V) and titanium (Ti) magnetite mine tailings found at Panzhihua in Sichuan, China, contain various harmful components, including heavy metals, most notably. The harsh environment has fostered the presence of beneficial microorganisms, such as plant-growth-promoting bacteria and heavy-metal-resistant microbes (Yu et al., 2014; Shen et al., 2023). However, there is no research on whether microbial strains with antagonistic effects on pathogens like *C. cassiicola* can be identified from among such tailings. Accordingly, this study was aimed at isolating antagonistic bacteria and elucidating their control effects on the pathogen *C. cassiicola* from the V-Ti magnetite tailings, providing biological control bacterial resources for kiwifruit leaf brown spot disease.

## Materials and methods

### Screening for antagonistic bacteria

**Figure 1:**
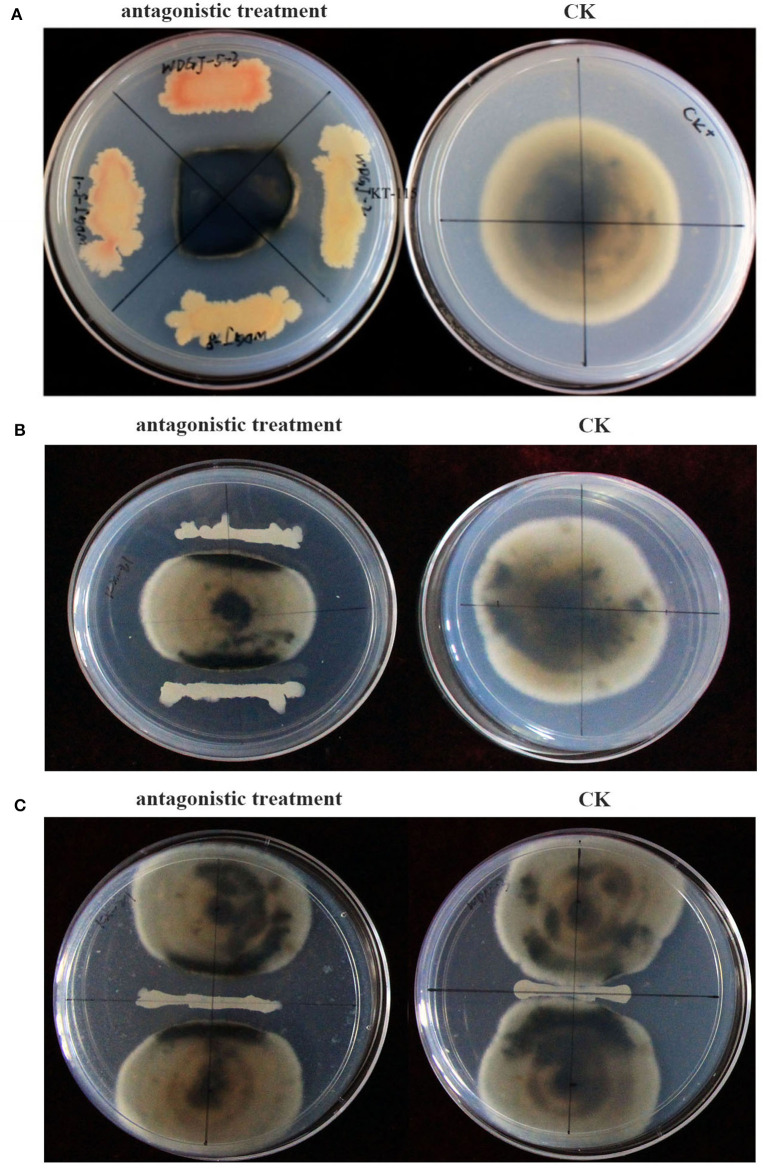
Antagonistic effects of the isolated bacterial strains against *Corynespora cassiicola* ACC10. **(A)** Screening method for antagonistic bacteria. **(B)** Determination of growth inhibitory rate (GIR). **(C)** Measurement of the antagonistic band (AB).

*Corynespora cassiicola* ACC10 (Cui et al., 2015), which was first identified as the pathogenic microorganism of the kiwifruit brown leaf spot, was used for confrontation culture. Additionally, a total of 136 bacteria (Yu et al., 2014) from V-Ti magnetite mine tailings at Panzhihua in Sichuan, China (26°36′59.8″N, 101°58′11.1″E) were used to preliminarily screen antagonistic strains by a confrontation culture method (Comby et al., 2017) on PDA medium (200 g/L potato, 10 g/L glucose, agar 18 g/L, and pH 7.4). Each PDA medium plate was divided into four equal sections (Figure 1A), and the isolated bacteria were streak inoculated with streak lengths of 2–3 cm. A 5-mm mycelial plug of *C. cassiicola* ACC10 was placed in the center of each PDA plate. Plates without streaked inoculation were used as blank controls. After incubation at 28°C for 5 days, the shapes of the pathogen colonies were observed, and the strains that significantly inhibited the growth of *C. cassiicola* ACC10 were identified as antagonistic.

### Quantitative evaluation of antagonistic effects of bacteria

The antagonistic effects of the screened antagonistic bacteria were determined by previously described growth inhibitory rate (GIR), antagonistic band (AB), and bacteriostatic rate (RE) assays (Bu et al., 2021; Zhang et al., 2022), with some modifications.

For the GIR assay, a 5-mm mycelial plug of the pathogenic fungus *C. cassiicola* ACC10 was inoculated at the center of a PDA plate, and antagonistic bacteria were streak inoculated on both sides (Figure 1B). Streaks of antagonistic bacteria strains were replicated three times, and pathogenic fungi without any antagonistic bacteria inoculation under the same culture conditions served as the control. After incubation at 28°C for 5 days, the diameters of the pathogen colony in each treatment (PTR) and the control (CKR) were measured, and the growth inhibitory rate (GIR) was calculated using the formula GIR(%) = (CKR – PTR)/CKR. A higher GIR indicated a stronger antagonistic effect.

For the AB assay, 2–3 cm streaks of antagonistic bacteria were inoculated in the center of PDA plates, and *C. cassiicola* ACC10 was inoculated at the center of each of the two halves of each plate (Figure 1C). The antagonistic band (AB) was determined as the distance between the closest edges of the two pathogen colonies. A higher AB indicated a stronger antagonistic effect. The antagonistic effect of each antagonistic bacteria strain was assessed in triplicate.

For the RE assay, 20 *C. cassiicola* strains (Supplementary Table S1) that can cause kiwi brown spots and were obtained from various disease outbreak sites in Sichuan or Chongqing were randomly selected to determine the bacteriostatic rate (RE) of 18 isolated antagonistic bacterial strains. The pathogenicity of the 20 *C. cassiicola* strains had been confirmed in previous research (Xu et al., 2023). RE was determined using the confrontation culture method of the GIR assay after cultivating them at the same temperature simultaneously. RE was defined as the percentage of antagonistic bacteria effectively controlling the 20 pathogenic *C. cassiicola* strains. A higher RE indicated a better control effect against the pathogenic fungi responsible for the kiwifruit brown leaf spot in Sichuan.

### Molecular identification of antagonistic bacteria

The total DNA of antagonistic bacteria was extracted by using the EZ-10 Spin Column Bacterial Genomic DNA Isolation Kit (Sangon Biotech, Shanghai, China), and stored at −20°C for molecular identification. All antagonistic bacteria were subjected to BOX-A1R clustering analysis, and then one representative strain per fingerprint type was chosen for 16S rRNA gene sequencing (Yu et al., 2014). The phylogenetic tree of the 16S rRNA sequences of antagonistic strains and reference strains was constructed using the neighbor-joining method with MEGA5.0 (Tamura et al., 2011). The 16S rRNA gene sequences of the representative strains were submitted to the GenBank database (accession numbers: KJ733949, KJ733981, KJ733954, KJ733993, KJ734012, KJ733995, KJ733985, KJ733947, KJ733955, KJ734004, KJ733963, KJ733996, and KJ733944).

### Antagonistic toxin production time of bacteria

Bacterial strains with a highly antagonistic effect were cultured in an LB liquid medium, and fresh culture filtrate was collected using disposable filters at 8-h intervals for the Oxford cup experiment (Zhou et al., 2022). Two sterilized Oxford cups were placed vertically on culture plates of *C. cassiicola* ACC10, positioned 3 cm away from the center (Figure 4B). To analyze the toxin production time of antagonistic bacteria, 200 μl of fresh filtrate collected at different culture periods were added to each Oxford cup, followed by incubation at 28°C for 2 days before recording the antagonistic effect. The diameter of the pathogen colony in the treatment (PTR) and the control (CKR) conditions were measured to obtain the growth inhibitory rate (GIR) of the antagonistic toxin of bacteria at different culture times.

### Control effect of antagonistic bacteria against kiwifruit brown spot in potted seedlings

The 2-year-old “Hongyang” kiwifruit seedlings were moved into a 25°C greenhouse. The control effect was determined after the seedlings had adapted to greenhouse conditions and their leaves had fully unfolded. Five-millimeter mycelial plugs of *C. cassiicola* ACC10 were placed on the leaves, with five mycelial plugs per leaf. After 40 h of culture, 50 ml culture broth diluent (10^7^ CFU/ml) of the antagonistic bacteria strain *Bacillus* sp. KT-10 was sprayed onto five leaves, and the same nutrient solution without antagonistic bacteria was sprayed on five leaves as the negative control. The experimental treatment and control were each repeated on three seedlings. The diameter of the brown spots was measured after 5, 10, 15, 20, 25, and 30 days. The differences in spot sizes were tested using a one-tailed paired-samples *t*-test.

### Control effect of antagonistic bacteria against kiwifruit brown spots in field conditions

An orchard with flat terrain, uniform fertility, and uniformly growing 8-year-old “Hongyang” kiwifruit vines was selected for the field experiment. *Bacillus subtilis* KT-10 fermentation broth (approximately 10^9^ cells/ml) was diluted 400 times and sprayed on kiwifruit leaves, while the medium was used as a negative control. The experiment treatments and control each consisted of three replicates, with a row of three kiwifruit trees in each replicate. The trees were sprayed at the initial time of kiwifruit brown spot occurrence, followed by additional spraying 7, 14, and 21 days later. Disease severity was assessed before spraying, 7 days after the first spraying, and 7 days after the last spraying. For disease severity assessment, the trees were sampled at five points, and 20 leaves were investigated at each time point. Disease severity was rated using a classification standard scale from 0 to 9 (Xu et al., 2023). Disease index (DI) and control effect (CE) were calculated according to the following formulas (Zhang et al., 2020):


$$\begin{array}{r} \text{DI}=\\ \frac{\sum (\text{the }{\text{leaves}}^{\text{′}}\text{number of every grade}\times \text{the value of disease grade})}{\begin{array}{r} \text{the number of all leaves}\times \text{the value of highest grade}\\ \times 100, \end{array}} \end{array}$$

$$\begin{array}{l} CE\left(\%\right)=\left(1-\frac{CK_{0}\times PT_{1}}{CK_{1}\times PT_{0}}\right)\times 100\%. \end{array}$$

where CK_0_ is the DI value of the control before spraying; CK_1_ is the DI value of the control after spraying; PT_0_ is the DI value of the treatment before spraying; and PT_1_ is the DI value of the treatment after spraying.

## Results

### Antagonistic bacteria isolated from V-Ti magnetite mine tailings

A total of 136 bacterial strains with different colony characteristics, including variations in the size, color, and morphology of colonies, were isolated from V-Ti magnetite mine tailings. Most of the strains were gram-positive, and their morphologies were predominantly rod-shaped (Yu et al., 2014). Among the 136 strains, only 18 strains exhibited the ability to dissolve the mycelia of *C. cassiicola* and form a boundary line on the PDA medium (Figure 1). Moreover, in the presence of these 18 strains, *C. cassiicola* colonies turned completely black, and the colonies were significantly smaller than those in the control (Figure 1). Thus, the 18 strains were considered antagonistic against *C. cassiicola*.

### Effectiveness evaluation of antagonistic bacteria

To accurately determine the antagonistic effect, two kinds of antagonistic culture assay methods were used for the 18 strains antagonistic to *C. cassiicola* ACC10. The 18 bacteria strains exhibited varying degrees of antagonistic effects on *C. cassiicola* ACC10. The growth inhibitory rate (GIR) values of the 18 bacteria strains ranged from 14.71 to 51.43% (Table 1), with strain KT-58 exhibiting the greatest inhibitory effect. The antagonistic band (AB) of 18 bacteria strains against *C. cassiicola* ACC10 ranged from 1.0 to 3.2 cm in width (Table 1). Strain KT-10 showed the widest AB, while that of KT-52 was the narrowest. The bacteriostatic rate (RE) test revealed that the 18 strains of antagonistic bacteria displayed varying bacteriostatic rates against 20 *C. cassiicola* strains, with RE values ranging from 45 to 100% (Table 1). Strains KT-115 and KT-10 exhibited the maximum bacteriostatic rate of 100%, and the RE values of both KT-71 and KT-60 were 90%. The RE of each of the other eleven strains was lower than 80%, with KT-121 having the lowest RE. Based on the three parameters estimated, strain KT-10 demonstrated a high antagonistic effect against *C. cassiicola*.

**Table 1:** Antagonistic effects of 18 tailings-isolated bacterial strains against *Corynespora cassiicola*.

**Strain**	**GIR (%)**	**AB (cm)**	**RE (%)**	**Strain**	**GIR (%)**	**AB (cm)**	**RE (%)**
KT-10	37.9 ± 1.5c	3.2 ± 0.4**a**	100 ± 0.0*a*	KT-60	26.3 ± 1.8ef	1.8 ± 0.1**cd**	90.1 ± 1.8*b*
KT-102	26.3 ± 1.2ef	1.8 ± 0.3**cd**	85.4 ± 1.4*c*	KT-64	26.8 ± 0.9e	1.8 ± 0.2**cd**	55.1 ± 1.9*i*
KT-103	25.0 ± 0.4ef	1.9 ± 0.4**bcd**	75.1 ± 2.9*e*	KT-65	23.8 ± 0.8fgh	1.9 ± 0.5**bcd**	59.9 ± 1.9*h*
KT-113	41.7 ± 1.1b	2.9 ± 0.3**a**	60.0 ± 3.4*h*	KT-70	16.0 ± 1.9j	1.9 ± 0.1**bcd**	69.7 ± 2.7*f*
KT-115	26.2 ± 2.2ef	2.3 ± 0.3**bc**	100 ± 0.0*a*	KT-71	20.5 ± 1.6i	2.0 ± 0.3**bcd**	90.3 ± 4.4*b*
KT-121	20.9 ± 2.1h	1.5 ± 0.2**de**	45.1 ± 1.7*k*	KT-72	21.4 ± 1.2gh	1.6 ± 0.2**d**	74.7 ± 1.8*e*
KT-52	25.0 ± 1.2ef	1.0 ± 0.2**e**	50.2 ± 2.0*j*	KT-83	18.2 ± 0.3i	2.4 ± 0.2**b**	85.2 ± 1.6*c*
KT-53	34.2 ± 1.4d	1.8 ± 0.2**cd**	50.2 ± 2.7*j*	KT-97	14.7 ± 0.6j	2.2 ± 0.3**bc**	80.2 ± 2.9*d*
KT-58	51.4 ± 3.4a	2.0 ± 0.2**bcd**	65.3 ± 2.8*g*	KT-99	25.0 ± 0.9ef	2.3 ± 0.4**bc**	69.8 ± 2.4*f*

GIR and AB were determined for *C. cassiicola* ACC10, and RE was tested using 20 pathogenic *C. cassiicola* strains.

GIR, growth inhibition rate; AB, antagonistic band; RE, bacteriostatic rate.

Values in the same column that are labeled with different letters were significantly different at *P*<*0.05*.

### Phylogeny of antagonistic bacteria in V-Ti magnetite mine tailings

**Figure 2:**
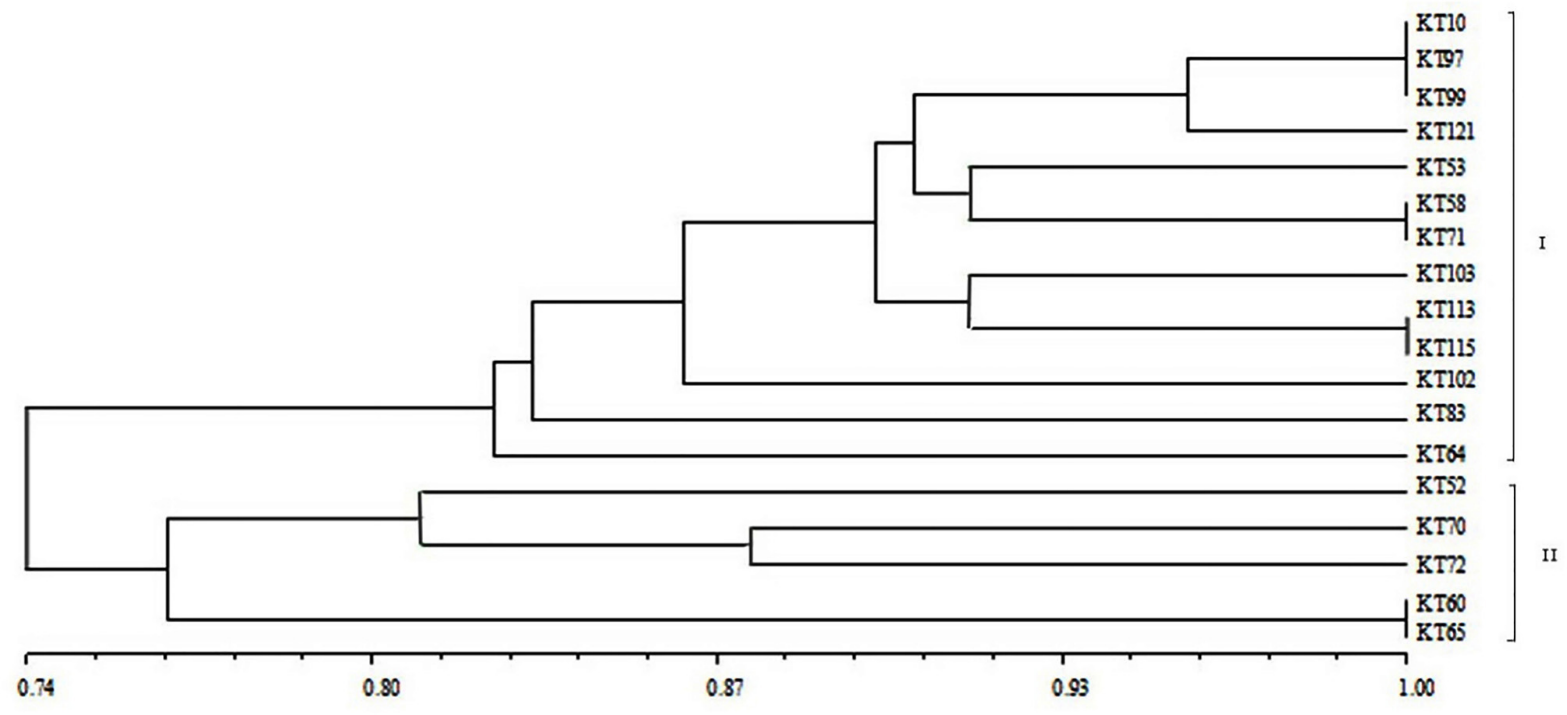
BOX-A1R cluster diagram of the antagonistic strains isolated from V-Ti magnetite mine tailings.

For BOX-A1R clustering fingerprint analysis, the 18 antagonistic strains were classified into two groups, labeled I and II, which contained 13 and five strains, respectively, at a 74% similarity level (Figure 2), indicating a diverse population of antagonistic bacteria was present in the mine tailings. Group I was further divided into five subgroups, while Group II was divided into two subgroups. The similarity among KT-10, KT-97, and KT-99 on one branch was 100%, and that between KT-58 and KT-71, between KT-113 and KT-115, and between KT-60 and KT-65 on three different branches was also 100%, respectively.

**Figure 3:**
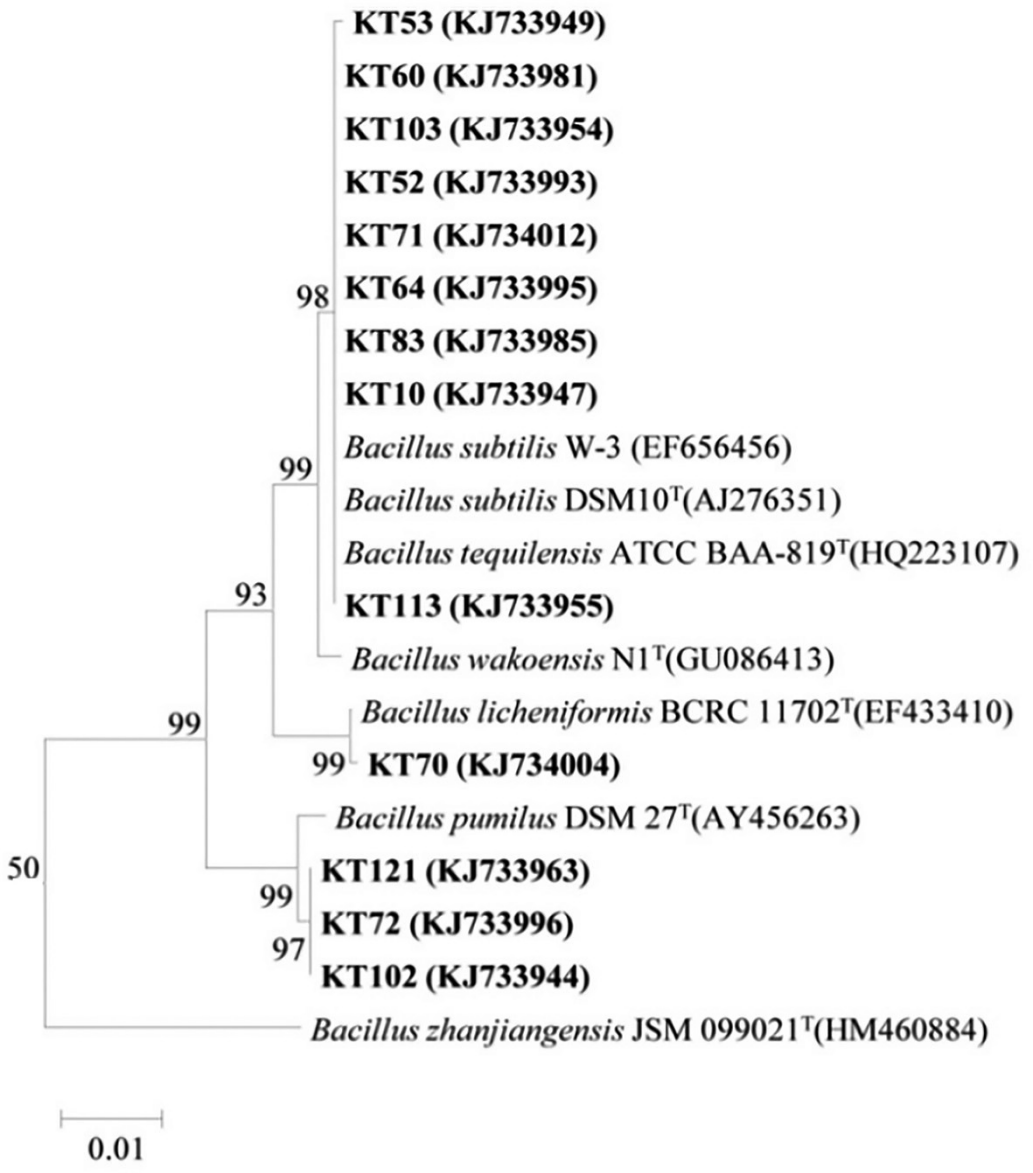
Phylogenetic tree generated from the 16S rRNA sequences of the representative antagonistic strains and reference strains by the neighbor-joining method.

Based on the BOX-A1R clustering tree, 13 representative antagonistic strains were selected at the 100% similarity level for 16S rRNA sequencing analysis. As BLASTn analysis of the GenBank database showed that the 16S rRNA sequences of the 13 strains were 99%−100% similar to the 16S rRNA sequences of *Bacillus*, the strains were preliminarily identified as *Bacillus* sp. The phylogenetic tree of the 16S rRNA sequences revealed that the 13 strains could be divided into three clades and thus belonged to different taxa (Figure 3). Among them, KT-53, KT-60, KT-103, KT-52, KT-71, KT-64, KT-83, KT-10, and KT-113 were located in the same clade as *Bacillus subtilis* and *Bacillus tequilensis*, indicating a high degree of homology and close genetic distance. Strains KT-70 and *Bacillus licheniformis* were in the same clade, and KT-121, KT-102, and KT-72 were in the same clade as *Bacillus pumilus*. However, it should be noted that using only 16S rRNA sequences may not enable the accurate identification of the species level for bacteria.

### Production time of bacterial antagonistic toxin

**Figure 4:**
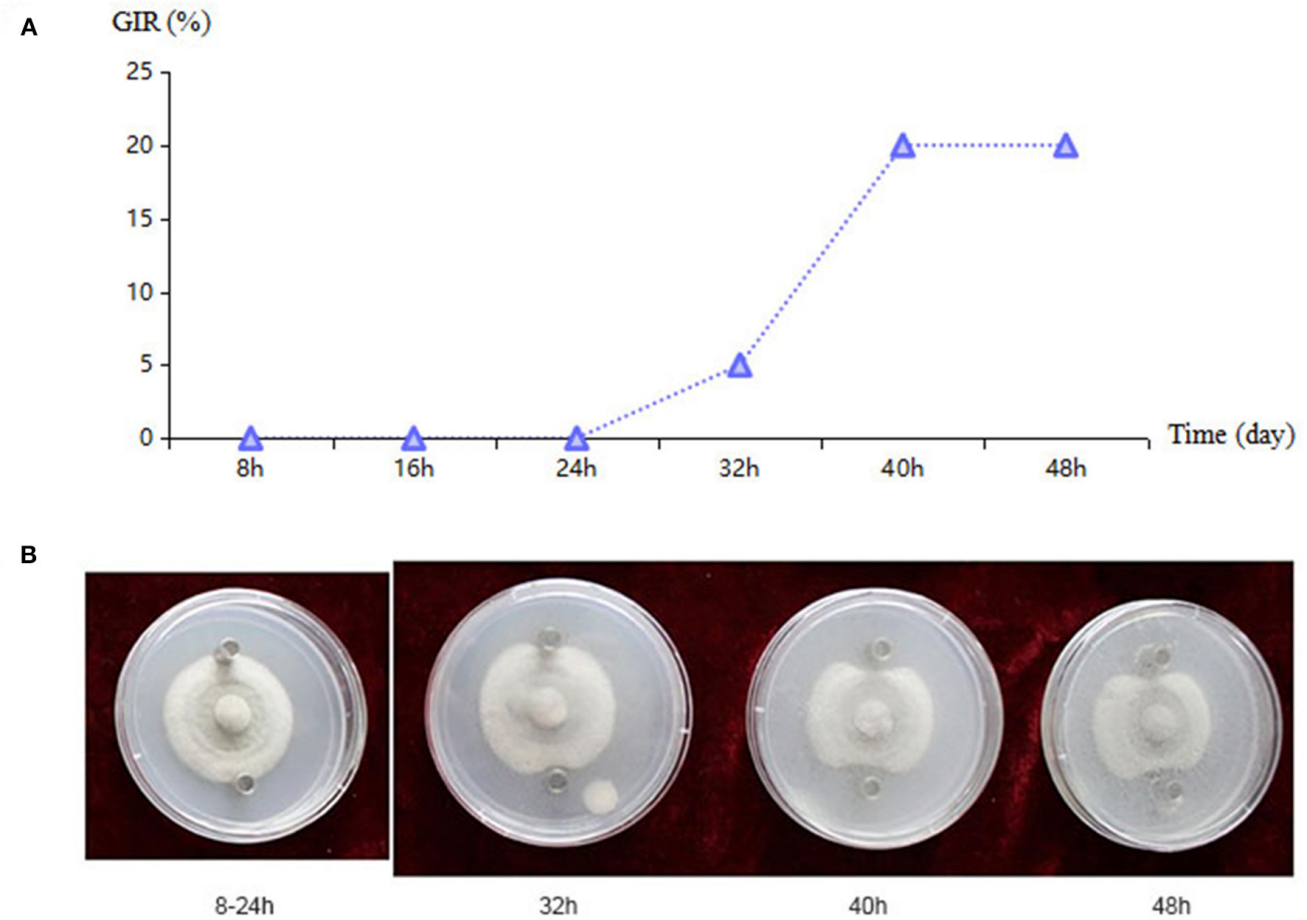
*Bacillus* sp. KT-10 antagonizing *Corynespora cassiicola* ACC10 in an Oxford cup experiment. **(A)** The growth inhibitory rate (GIR) for the antagonistic toxin of bacteria at different culture times. **(B)** Antagonistic effect of KT-10 toxin as observed in a time series of images of the Oxford cup experiment.

The strains KT-10, KT-60, KT-71, KT-83, and KT-115 displayed strong antagonistic effects and were thus selected to analyze the time of antagonistic toxin production (Supplementary Table S2). Strains KT-10 and KT-115 began producing antagonistic toxins after 32 h of culture and continued to increase toxin production after 40 h (Figure 4A). Strains KT-60 and KT83 also started producing toxins after 32 h, but their toxin levels did not increase after 40 h, and the toxicity of KT-60 disappeared by 48 h. KT-71 began toxin production at 40 h, but there was no increase at 48 h. Thus, there was substantial diversity in the antagonistic toxin production time among the strains. KT-10 exhibited a strong inhibition effect on the spread of kiwifruit brown spots, effectively preventing and controlling the expansion of the kiwifruit brown leaf spot disease pathogen. The toxin secretion by KT-10 and its antifungal effect was enhanced with prolonged culture duration (Figure 4B).

### Control effect of KT-10 on kiwifruit brown leaf spots in greenhouse conditions

**Figure 5:**
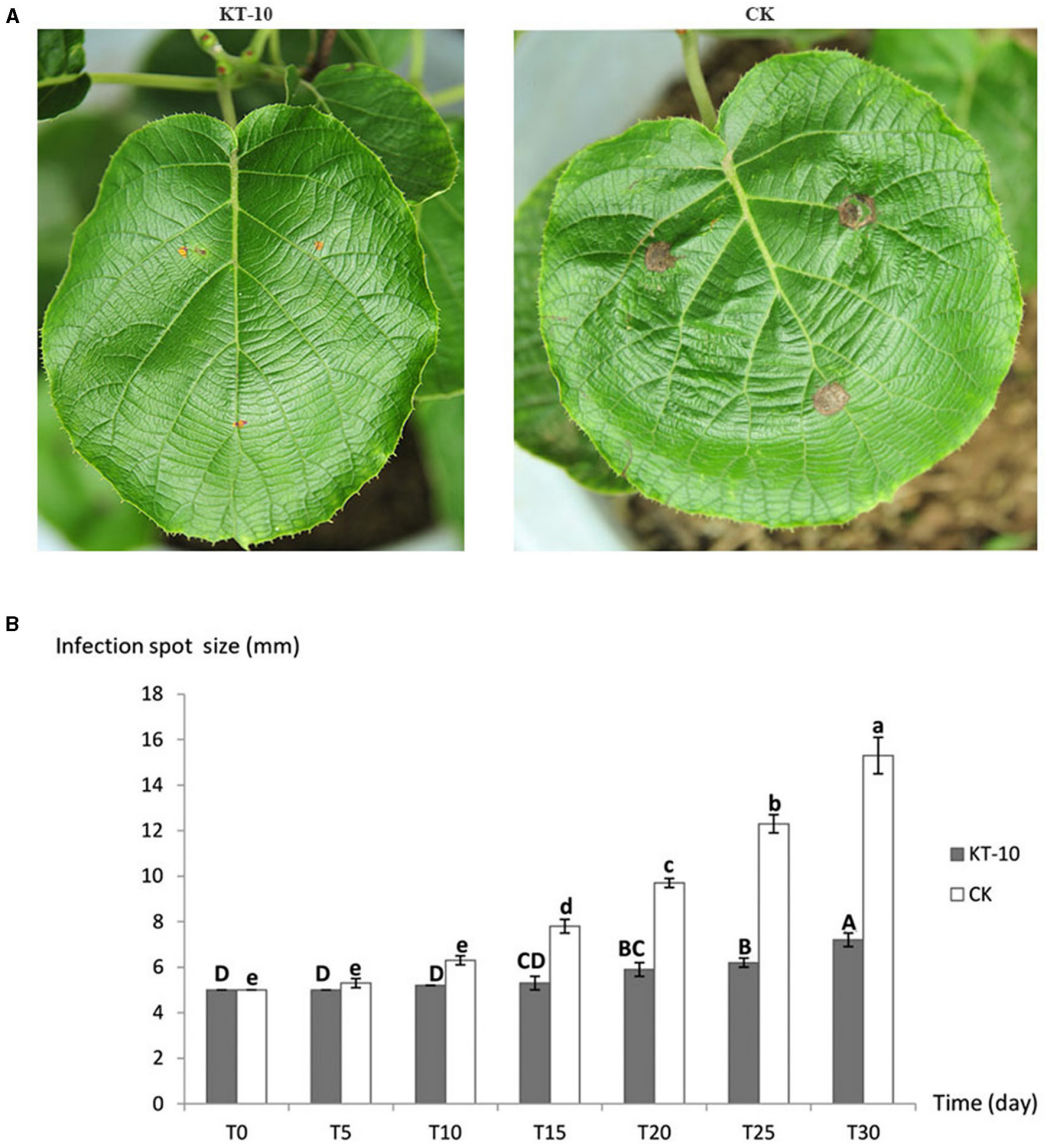
Control effect of *Bacillus* sp. KT-10 on the spread rate of *Corynespora cassiicola* ACC10 on kiwifruit leaves in the pot experiment. **(A)** Images showing the typical control effect for kiwifruit brown leaf spot on a plant grown in a pot. **(B)** Infection spot size at different times. Different letters indicate a significant difference (*p* < 0.05) among different infection time.

Based on the assays of antagonistic effectiveness and antagonistic toxin production time, KT-10, the strain with the strongest antagonistic effect, was selected to characterize its control effect on the kiwifruit brown leaf spot disease caused by *C. cassiicola* ACC10. The brown spots on kiwifruit leaves began to expand 10 days after being sprayed with the strain KT-10, while in the control (CK) conditions without antagonistic bacteria, the spots began to expand just 5 days after spraying. The spread rate of brown spots in the KT-10 treatment was slow, with an average spot size of only 7.2 mm in diameter, which is 1.44 times that of the initial ACC10 mycelial plugs on day 30 (Figure 5B). In contrast, the brown spots in the CK treatment rapidly expanded to 15.3 mm on day 30, reaching a diameter more than three times the initial 5-mm diameter of the mycelial plugs of the ACC10 (Figure 5B). Thus, strain KT-10 had a substantial control effect on kiwifruit brown leaf spots (Figure 5A).

### Control effect of KT-10 on kiwifruit brown leaf spots in field conditions

**Figure 6:**
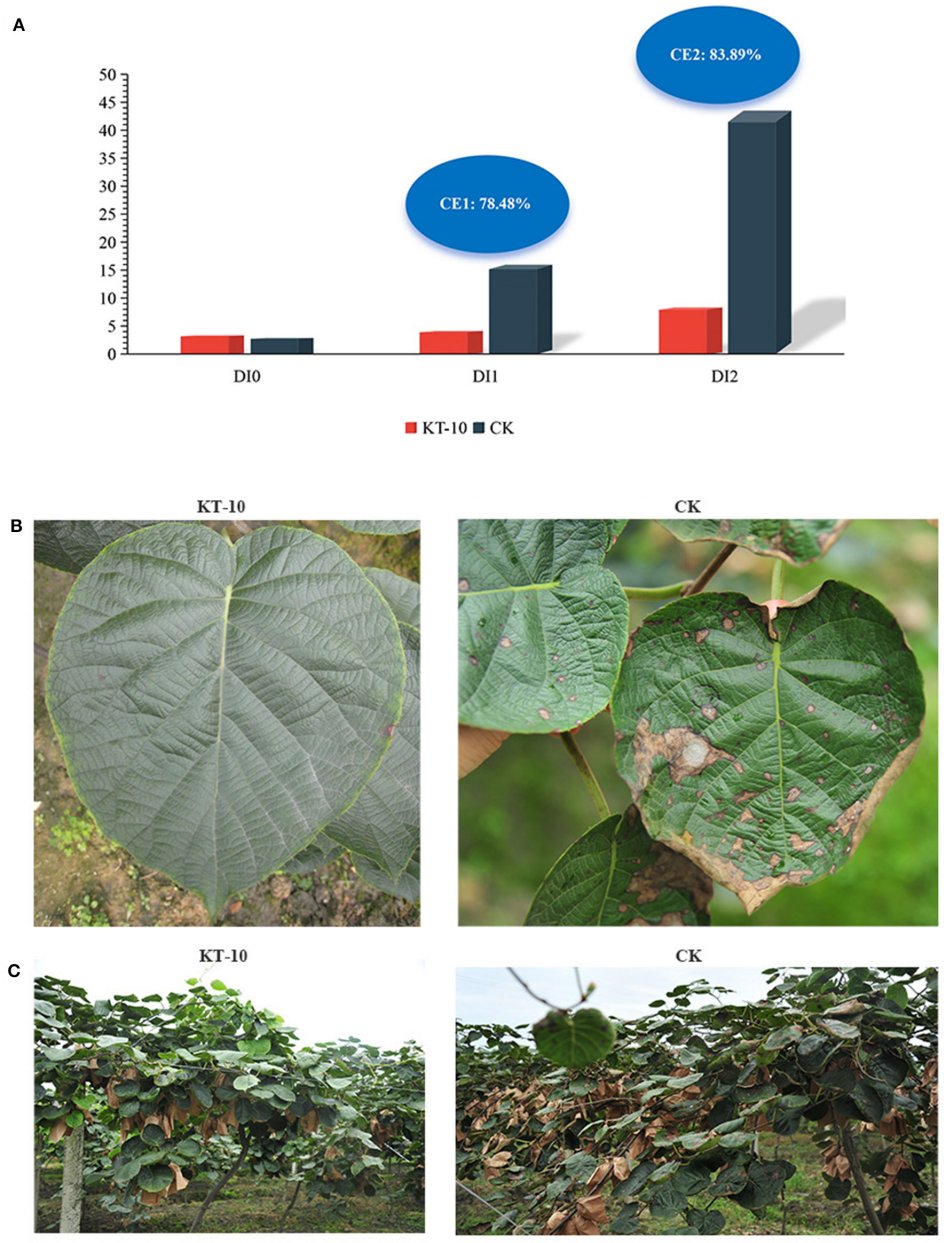
Control effect of *Bacillus* sp. KT-10 on the brown leaf spot of kiwifruit in the field. **(A)** Disease index (DI) and control effect (CE) of the kiwifruit brown spot disease. **(B)** Image showing the typical control effect of kiwifruit brown leaf spots in the field. **(C)** Field control effect of kiwifruit brown leaf spot. DI0, DI value before spraying KT-10; DI1, DI value at 7 days after first spraying KT-10; DI2, DI value at 7 days after last spraying KT-10; CE1, CE value at 7 days after first spraying KT-10; CE2, CE at 7 days after the last spraying of KT-10.

To further assess the control effect of *Bacillus* sp. KT-10 on kiwifruit brown leaf spots, the strain was applied to kiwifruit in field conditions. After 7 days of the first spraying, the disease index (DI) of kiwifruit brown leaf spots in the KT-10 treatment had increased from the initial value of 3.36–4.08; meanwhile, DI had increased from 2.82 to 15.91 in the CK treatment, and the relative control effect of KT-10 was 78.48% (Figure 6A). Seven days after the last spraying, DI was 8.36 in the KT-10 treatment and 43.54 in the CK treatment; therefore, the relative control effect of KT-10 was 83.89%. Thus, the kiwifruit brown leaf spot disease became more severe without any control measures. However, *Bacillus* sp. KT-10 effectively controlled the disease in the field (Figures 6B, C).

## Discussion

Plant diseases pose a serious threat to agricultural production, and the primary approach to their prevention and control has traditionally been the application of chemical pesticides. However, the long-term and extensive use of chemical pesticides has led to issues such as pesticide residues, environmental pollution, human health consequences, and the disruption of ecological balance (Lata et al., 2021). Accordingly, biological control methods have gained widespread acceptance and application owing to their advantages, including highly reduced environmental pollution, safety, an absence of chemical product residues, high specificity to pathogens, avoidance of pesticide resistance, and high efficiency (Pérez-Sánchez et al., 2018). Beneficial microorganisms, such as *Bacillus, Pantoea, Streptomyces, Trichoderma, Clonostachys, Pseudomonas, Burkholderia*, certain yeasts, and their metabolites, are primarily employed in biological control agents (Lahlali et al., 2022).

Some beneficial bacteria with effects that include plant growth promotion, resistance enhancement, and bioremediation of heavy metals have previously been isolated from V-Ti magnetite mine tailings, which are characterized by their extreme conditions of heavy metal contamination and nutrient scarcity (Yu et al., 2014, 2017). Extremophiles are relevant to a broad range of fields, spanning biotechnology, biodegradation, bioremediation, biorefining, and astrobiology, as well as various industries, such as agriculture and the production of pharmaceuticals, food, cosmetics, and textiles (Kochhar et al., 2022). V-Ti magnetite mine tailings can harbor an abundance of plant-growth-promoting microorganisms, including nitrogen-fixing rhizobia and indoleacetic acid-producing, siderophore-secreting, and heavy metal-tolerant microbes (Yu et al., 2014; Shen et al., 2023). In the present study, 18 bacterial strains isolated from the V-Ti magnetite mine tailings displayed various antagonistic effects on kiwifruit brown spot disease. This indicates that a diverse population of beneficial bacteria with antagonistic effects can thrive in extreme environments such as V-Ti magnetite mine tailings. Among these 18 antagonistic bacterial strains, KT-10 demonstrated substantial antagonistic and control effects and could potentially serve as a biocontrol agent for kiwifruit brown spot disease.

The kiwifruit leaf brown spot disease is a severe issue in both Sichuan Province and Guangxi Zhuang Autonomous Region, with *C. cassiicola* as the predominant pathogen (Yuan et al., 2014; Cui et al., 2015). *Corynespora cassiicola* infects a wide range of host species and has diverse transmission routes, making rapid and effective control measures essential for agricultural production (Shimomoto et al., 2011). In severe cases, *C. cassiicola* can infect the flowers, fruits, and stems (Qi et al., 2009). *Corynespora cassiicola* was even observed to infect soybeans [*Glycine max* (L.) Merr.], extracting nutrients from leaves, stems, pods, seeds, and roots or surviving in an endophytic relationship with soybeans (Edwards Molina et al., 2022).

Various naturally occurring microorganisms have been isolated that are capable of antagonizing or eradicating pathogenic microorganisms to achieve biological control of diseases. Biocontrol microorganisms work through mechanisms that include competition, antagonism, and the stimulation of plant defense mechanisms against pathogens, thereby inducing plant resistance mechanisms (Zehra et al., 2021). The most effective bioagents studied are those that employ multiple mechanisms, as seen in *Pseudomonas*, which utilizes both antibiosis and the induction of host resistance to suppress disease-causing microorganisms (Junaid et al., 2013). Genera such as *Bacillus* and *Pseudomonas* and their bioactive secondary metabolites have been considered beneficial bio-controllers of plant diseases through direct antibiosis, plant growth promotion, and the induction of systemic resistance in plant hosts (Dimkić et al., 2022). Bacteriophages, actinomycetes, and *Bacillus* spp. have shown biocontrol potential against *P. syringae*, the pathogen responsible for kiwifruit bacterial canker disease (Tu et al., 2011; Biondi et al., 2012; Frampton et al., 2014). However, the resources for bio-control microorganisms against kiwifruit leaf spot remain limited. In the present study, 18 bacterial strains among the 136 strains isolated from V-Ti magnetite tailings were antagonistic to kiwifruit leaf brown spot disease. The rare antagonistic bacteria in the V-Ti magnetite mine tailings could be one of the reasons for the prolonged absence of plant growth in that area. However, differences in the antagonistic effects of the 18 bacteria strains were observed, despite the strains all belonging to the genus *Bacillus*.

The utilization of *Bacillus* for biological control of plant diseases has been an active research area (Comby et al., 2017). Among the extensively studied *Bacillus* species, *B. subtilis* is well documented for its roles as a growth promoter and antagonist of various pathogens. The disease suppression mechanisms employed by *B. subtilis* include plant growth promotion, antibiosis, competition for space and nutrients, lysis of pathogen hyphae, and the induction of systemic resistance (Wang X. Q. et al., 2018). For example, *B. subtilis* (at 10^8^ CFU/ml) exhibited a remarkable 68.20% inhibition rate against spore germination of *Hemileia vastatrix*, the causative agent of coffee rust (Daivasikamani, 2009). *Bacillus* species have also been explored as a biological tool for enhancing crop performance across adverse environments through the induction of biomolecular changes (Radhakrishnan et al., 2017). In this study, 13 representative antagonistic bacterial strains from V-Ti magnetite mine tailings were identified as *B. licheniformis*, three strains as *B. pumilus*, and seven strains as *B. subtilis* or *B. tequilensis*. *Bacillus tequilensis* is closely related to *B. subtilis*, obstructing the positive identification of strains as belonging to one species or the other based on 16S rRNA sequence analysis alone (Gatson et al., 2006). Moreover, sequence similarity of at least 99% in 16S rRNA full-length sequences has been previously recommended as a genus-level threshold (Robert, 2018). Hence, all of the representative antagonistic strains were determined to belong to the genus *Bacillus*, which is characterized by its strong adaptability and resistance to heavy metals and barren environments (Yu et al., 2014).

*Corynespora cassiicola*, as a broad-spectrum pathogen, can cause spot disease in many host plant species. However, several microorganisms have been found to antagonize or control this pathogen. For example, a strain of *Meyerozyma caribbica* isolated from *C. cassiicola*-infected soybean plants inhibited *C. cassiicola* mycelial growth by producing antifungal phenethyl alcohol, suggesting a novel biocontrol method for managing target spot disease in soybean (Zhang et al., 2023). *Trichoderma spirale*, through producing antifungal alcohols and pyran compounds, displayed biocontrol activity against leaf spot disease caused by *C. cassiicola* in lettuce (*Lactuca sativa* L.) (Baiyee et al., 2019). In the present study, a toxin production test showed that the strain KT-10 produced toxin at 32 h, with toxin production gradually increasing at both 40 and 48 h. This suggests that KT-10 can produce toxins during its growth stages to inhibit the growth of *C. cassiicola*, which affects crops beyond kiwifruit. *Corynespora cassiicola* can cause target spot diseases in cotton (*Gossypium hirsutum* L.) and soybean, with isolates from a single location in Tennessee including eight unique multilocus genotypes, reflecting the genetic diversity of C. *cassiicola* isolates (Rondon and Lawrence, 2021). In this study, we used antagonistic bacteria against 20 strains of *C. cassiicola* randomly selected from 20 different disease outbreak sites throughout Sichuan to assess the antagonistic effect of KT-10 against different pathogen genotypes. The varying bacteriostatic rates also indicate that the 20 strains of *C. cassiicola* from different sites in Sichuan exhibit meaningful phenotypic diversity (Xu et al., 2023). The observed 100% bacteriostatic rate (RE) also showed that *Bacillus* sp. KT-10 had relatively broad control against *C. cassiicola*.

Despite the relatively common occurrence of kiwifruit leaf brown spots in Sichuan, effective prevention and control measures remain elusive. Recently, a maize biochar-zinc oxide (MB-ZnO) nanocomposite was considered an environmentally friendly method for managing kiwifruit leaf spot disease, achieving the highest growth inhibition (79%) at a 19 mg/ml nanoparticle dose (Kamal et al., 2023). Biological control, especially using antagonistic microorganisms, has become a widely adopted strategy in the control of plant diseases. Among the 18 antagonistic strains isolated from V-Ti magnetite mine tailings in the present study, KT-10 demonstrated the most pronounced growth inhibition against the 20 pathogenic *C. cassiicola* strains assayed, reaching a rate of 100% bacteriostatic rate (RE), with an antagonistic bandwidth of up to 3.2 cm and an inhibitory rate of up to 100%. Therefore, *Bacillus* sp. KT-10 can be considered an effective antagonistic microbe against *C. cassiicola*. Moreover, the inhibitory effects of strain KT-10 on kiwifruit brown spots were verified in both pot and field experiments. The strain KT-10 exhibited significant effects in the assessment of kiwifruit brown spot control in the pot experiment, inhibiting and preventing the expansion of kiwifruit brown spot symptoms. Although the DI slightly increased after KT-10 application in the field experiment, the control effect reached 84% after the third application, far surpassing the control treatment in terms of reduced symptom onset at 30 days. This demonstrated that *B. subtilis* KT-10 can effectively control kiwifruit brown spots in the field, establishing its potential application in brown spot disease management.

## Conclusion

We identified several bacterial strains, previously isolated from V-Ti magnetite mine tailings, which exhibited antagonistic effects against the pathogenic fungus *C. cassiicola*, the causal agent of kiwifruit brown leaf spot disease. All of these strains were identified as belonging to the *Bacillus* genus. However, these antagonistic bacteria showed varying control effects and toxin production times. Among them, strain KT-10 showed the most significant effect on kiwifruit brown leaf spot disease and therefore has the potential to be a biocontrol agent. Moreover, the present results indicate that biological control microorganisms can be found in extreme soil environments such as V-Ti magnetite mine tailings. The present results suggest the application of KT-10 in kiwifruit production could represent a breakthrough in brown spot disease control.

## Data availability statement

The datasets presented in this study can be found in online repositories. The names of the repository/repositories and accession number(s) can be found at: https://www.ncbi.nlm.nih.gov/genbank/, KJ733949, KJ733981, KJ733954, KJ733993, KJ734012, KJ733995, KJ733985, KJ733947, KJ733955, KJ734004, KJ733963, KJ733996, KJ733944.

## Author contributions

YC: Writing—review & editing, Writing—original draft, Data curation, Formal analysis, Investigation, Methodology, Visualization. YZ: Data curation, Formal analysis, Investigation, Methodology, Visualization, Writing—original draft. GD: Data curation, Formal analysis, Investigation, Methodology, Writing—review & editing. YL: Data curation, Formal analysis, Investigation, Methodology, Writing—review & editing. JX: Data curation, Formal analysis, Investigation, Methodology, Writing—review & editing. ZC: Investigation, Writing—review & editing. LL: Investigation, Writing—review & editing. GG: Writing—review & editing, Supervision. XY: Supervision, Writing—review & editing, Data curation, Formal analysis, Investigation, Project administration, Writing—original draft.
