# Supplementary material for: Evaluation of the control efficacy of antagonistic bacteria from V-Ti magnetite mine tailings on kiwifruit brown spots in pot and field experiments
Source: Front Microbiol. 2024 Mar 12;15:1280333. doi: 10.3389/fmicb.2024.1280333 (PMC10963537; doi:10.3389/fmicb.2024.1280333)
Supplement: Supplementary file 1 [file Table_1.DOCX]

Table S1 pathogenicity and sources of 20 *Corynespora cassiicola* strains to kiwifruit

| Strain | Host | Disease index (DI)^x^ | Sources Location |
| --- | --- | --- | --- |
| CQ2 | kiwifruit | 30.86±4.28(H) | Qianjiang, Chongqin Municipality |
| DJY1 | kiwifruit | 40.74±0.00(H) | Dujiangyang, Chengdu, SC |
| DJY22 | kiwifruit | 28.40±4.28(H) | Dujiangyang, Chengdu, SC |
| DJY32 | kiwifruit | 33.33±0.00(H) | Dujiangyang, Chengdu, SC |
| DY1 | kiwifruit | 20.99±4.28(M) | Mianzhu, Deyang, SC |
| DY5 | kiwifruit | 16.05±2.14(L) | Shifang, Deyang, SC |
| EM2 | kiwifruit | 25.93±0.00(H) | E'mei, Leshan, SC |
| GY3 | kiwifruit | 28.40±4.28(H) | Cangxi, Guangyuan, SC |
| HB2 | kiwifruit | 30.86±4.28(H) | Chibi, Xian'ning, HB |
| MY10 | kiwifruit | 20.99±4.28(M) | Anzhou, Mianyang, SC |
| MY2 | kiwifruit | 20.99±4.28(M) | Beichuan, Mianyang, SC |
| PJ1 | kiwifruit | 25.93±0.00(H) | Pujiang, Chengdu, SC |
| PJ9 | kiwifruit | 23.46±4.28(M) | Pujiang, Chengdu, SC |
| QL2 | kiwifruit | 35.80±4.28(H) | Qionglai, Chengdu, SC |
| QL4 | kiwifruit | 23.46±4.28(M) | Qionglai, Chengdu, SC |
| QL8 | kiwifruit | 28.40±4.28(H) | Qionglai, Chengdu, SC |
| SL5 | kiwifruit | 28.40±4.28(H) | Shuangliu, Chengdu, SC |
| YA21 | kiwifruit | 35.80±8.55(H) | Yingjing, Ya'an, SC |
| YA25 | kiwifruit | 13.58±4.28(L) | Yucheng, Ya'an, SC |
| YA5 | kiwifruit | 28.40±4.28(H) | Mingshan, Ya'an, SC |
