# Supplementary material for: Evaluation of the control efficacy of antagonistic bacteria from V-Ti magnetite mine tailings on kiwifruit brown spots in pot and field experiments
Source: Front Microbiol. 2024 Mar 12;15:1280333. doi: 10.3389/fmicb.2024.1280333 (PMC10963537; doi:10.3389/fmicb.2024.1280333)
Supplement: Supplementary file 2 [file Table_2.docx]

SUPPLEMENTATY TABLE 2 Antagonistic effect of the culture filtrates of the strains cultured for different time against *Corynespora cassiicola* ACC10

| Bacteria | 8h | 16h | 24h | 32h | 40h | 48h |
| --- | --- | --- | --- | --- | --- | --- |
| KT-10 | - | - | - | + | ++ | ++ |
| KT-115 | - | - | - | + | ++ | ++ |
| KT-60 | - | - | - | + | + | - |
| KT-71 | - | - | - | - | + | + |
| KT-83 | - | - | - | + | + | + |

-, no bacteriostatic ring; +,bacteriostatic ring < xx ; ++, bacteriostatic ring > xx.
